# quarTeT: a telomere-to-telomere toolkit for gap-free genome assembly and centromeric repeat identification

**DOI:** 10.1093/hr/uhad127

**Published:** 2023-06-13

**Authors:** Yunzhi Lin, Chen Ye, Xingzhu Li, Qinyao Chen, Ying Wu, Feng Zhang, Rui Pan, Sijia Zhang, Shuxia Chen, Xu Wang, Shuo Cao, Yingzhen Wang, Yi Yue, Yongsheng Liu, Junyang Yue

**Affiliations:** College of Life Science, Sichuan University, Chengdu, Sichuan 610064, China; School of Horticulture, Anhui Agricultural University, Hefei, Anhui 230036, China; School of Information and Computer, Anhui Agricultural University, Hefei, Anhui 230036, China; State Key Laboratory of Tea Plant Biology and Utilization, Anhui Agricultural University, Hefei, Anhui 230036, China; School of Horticulture, Anhui Agricultural University, Hefei, Anhui 230036, China; School of Horticulture, Anhui Agricultural University, Hefei, Anhui 230036, China; School of Horticulture, Anhui Agricultural University, Hefei, Anhui 230036, China; School of Horticulture, Anhui Agricultural University, Hefei, Anhui 230036, China; School of Horticulture, Anhui Agricultural University, Hefei, Anhui 230036, China; School of Horticulture, Anhui Agricultural University, Hefei, Anhui 230036, China; School of Information and Computer, Anhui Agricultural University, Hefei, Anhui 230036, China; State Key Laboratory of Tea Plant Biology and Utilization, Anhui Agricultural University, Hefei, Anhui 230036, China; Agricultural Genomics Institute at Shenzhen, Chinese Academy of Agricultural Sciences, Shenzhen, Guangdong 518124, China; Agricultural Genomics Institute at Shenzhen, Chinese Academy of Agricultural Sciences, Shenzhen, Guangdong 518124, China; Key Laboratory of Horticultural Plant Biology Ministry of Education, Huazhong Agricultural University, Wuhan, Hubei 430070, China; School of Horticulture, Anhui Agricultural University, Hefei, Anhui 230036, China; School of Information and Computer, Anhui Agricultural University, Hefei, Anhui 230036, China; State Key Laboratory of Tea Plant Biology and Utilization, Anhui Agricultural University, Hefei, Anhui 230036, China; College of Life Science, Sichuan University, Chengdu, Sichuan 610064, China; School of Horticulture, Anhui Agricultural University, Hefei, Anhui 230036, China; State Key Laboratory of Tea Plant Biology and Utilization, Anhui Agricultural University, Hefei, Anhui 230036, China; School of Horticulture, Anhui Agricultural University, Hefei, Anhui 230036, China; Agricultural Genomics Institute at Shenzhen, Chinese Academy of Agricultural Sciences, Shenzhen, Guangdong 518124, China

## Abstract

A high-quality genome is the basis for studies on functional, evolutionary, and comparative genomics. The majority of attention has been paid to the solution of complex chromosome structures and highly repetitive sequences, along with the emergence of a new ‘telomere-to-telomere (T2T) assembly’ era. However, the bioinformatic tools for the automatic construction and/or characterization of T2T genome are limited. Here, we developed a user-friendly web toolkit, quarTeT, which currently includes four modules: AssemblyMapper, GapFiller, TeloExplorer, and CentroMiner. First, AssemblyMapper is designed to assemble phased contigs into the chromosome-level genome by referring to a closely related genome. Then, GapFiller would endeavor to fill all unclosed gaps in a given genome with the aid of additional ultra-long sequences. Finally, TeloExplorer and CentroMiner are applied to identify candidate telomere and centromere as well as their localizations on each chromosome. These four modules can be used alone or in combination with each other for T2T genome assembly and characterization. As a case study, by adopting the entire modular functions of quarTeT, we have achieved the *Actinidia chinensis* genome assembly that is of a quality comparable to the reported genome Hongyang v4.0, which was assembled with the addition of manual handling. Further evaluation of CentroMiner by searching centromeres in *Arabidopsis thaliana* and *Oryza sativa* genomes showed that quarTeT is capable of identifying all the centromeric regions that have been previously detected by experimental methods. Collectively, quarTeT is an efficient toolkit for studies of large-scale T2T genomes and can be accessed at http://www.atcgn.com:8080/quarTeT/home.html without registration.

## Introduction

The quality of genome assembly determines the effectiveness of functional, evolutionary, and any other downstream analysis. Besides the protein-coding gene regions, accumulating studies have shown that repetitive sequences can impose significant effects on gene expression [1]. Among the highly repetitive regions, centromere and telomere have been brought to attention because of their important functions in cell division and chromosomal replication [2]. With the development of third-generation sequencing (TGS) technologies such as PacBio HiFi [3] and ONT ultra-long [4], the complete assembly of highly repetitive regions has become possible, and the cost has also decreased to an acceptable level for the majority of researchers [5]. Several bioinformatic tools such as minimap2 [6] and hifiasm [7] have been developed and widely used to handle TGS datasets in recent genomic studies. A new ‘telomere-to-telomere (T2T) assembly’ era is opening, allowing us to further understand the characteristics and evolution of highly repetitive regions in an increasing number of genomes [8–12]. Many researchers are making efforts to catch up with the new era, which in turn stimulates the demand for T2T reference genomes. In particular, this requires cutting-edge tools to assemble and analyse multiple high-quality genomes efficiently. However, few tools are able to build a workflow for this time-consuming work.

Centromere structure is a highlighted topic in the studies of T2T genome [13]. Unfortunately, due to its highly variable and rapidly evolving characteristics, centromeric identification is still a difficult issue [14]. Although PFGE and ChIP-seq have been proven reliable in locating centromere [15, 16], their instruments and reagents required by the experimental methods are hard to access for scientists inexperienced in this aspect. Additionally, these assays do not suit large-scale genomic studies. Tandem Repeat Finder (TRF) can be used to search possible centromeric repeats in genome sequences [17], but it is not designed to focus on the centromere. Sometimes it is hard to distinguish centromeric repeats from other tandem repeats.

In this study, we developed quarTeT, a user-friendly web toolkit specially designed for T2T genome assembly and characterization, including reference-guided genome assembly, ultra-long sequence-based gap filling, telomere identification, and *de novo* centromere prediction. The quarTeT is named by the abbreviation ‘Telomere-To-Telomere Toolkit’ (TTTT), representing the combination of four modules. It also integrates the first tool to predict centromere candidates independent of the experimental evidence or previous centromeric studies. We have assembled and characterized the primary haplotype of *Actinidia chinensis* cv. ‘Hongyang’ genome assembly (hereafter named HY4Q), then compared it with our previously published manual assembly HY4P [18] to evaluate the program performance. Furthermore, we have examined our *de novo* centromere prediction module by identifying the centromeres and comparing them with experimental evidence in *Arabidopsis thaliana* [19] and *Oryza sativa* [20] genomes. The results demonstrate that our toolkit supports high-quality genome assembly for T2T genomic studies, even for pan-genomic studies, in an automatic workflow. Meanwhile, the command-line program of our toolkit is also available, which allows multiple processes and further advanced options. The quarTeT toolkit can be freely accessed at http://www.atcgn.com:8080/quarTeT/home.html.

## Results

### Program description

**Figure 1:**
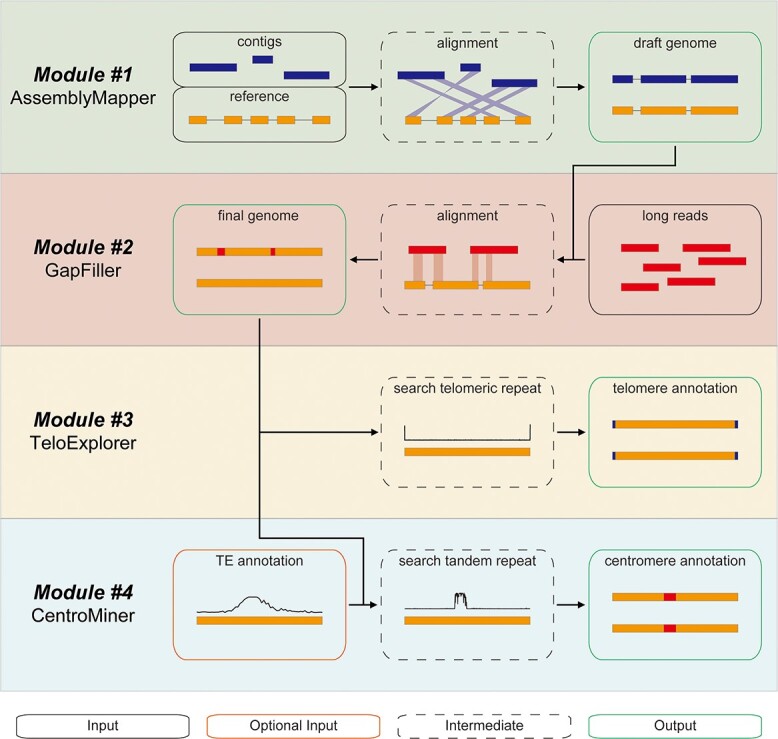
The overall workflow of quarTeT. The quarTeT toolkit provides four modules, which can be used alone or in combination to assemble and/or characterize a T2T genome. The input files are illustrated in a solid black box, and the optional input files are drawn in the orange box. Intermediate operations are shown in the dotted line box, and output files are presented in the green box. When used in combination, the output of a module can be used as the input of the other modules.

The workflow of quarTeT is illustrated in Fig. 1. Briefly, quarTeT currently includes four modules: AssemblyMapper, GapFiller, TeloExplorer, and CentroMiner. These four modules can be used alone or in combination with each other for the assembly and characterization of T2T genomes. As an advanced feature, the quarTeT toolkit also supports the command line interface. When used via the command line, quarTeT can run in multiple processes, hence having a speed boost in compute cluster. Further advanced options like detailed alignment arguments are also available. It also provides convenience to build pipelines with quarTeT modules. This should be helpful to the research of multiple genomes like the pan-genomic study.

#### Module #1: AssemblyMapper

AssemblyMapper assembles contigs into pseudo-chromosome with the guide of a reference genome. This module takes a phased contig level assembly and a closely related reference genome as input, both in FASTA format. It is recommended to get such phased assembly via hifiasm software [7] using long reads generated from the PacBio HiFi platform. First, the input contigs are examined. If any gaps are found, these contigs will be broken at gaps. Then, the contigs shorter than the given length will be removed. Telomeres in contigs are also identified to improve the assembly. Next, alignments are generated between contigs and the reference genome. After filtering the low-quality alignment, contigs with no alignment are discarded, and the remaining contigs are assigned to the best matches chromosome in the reference genome. Finally, the contigs assigned to each chromosome are sorted based on reference coordinates, and 100× N are placed between adjacent contigs, generating pseudo-chromosomes. In addition, this module generates useful statistical data such as contigs’ destinations, total placed and unplaced bases, AGP format assembly description, and gap locations.

#### Module #2: GapFiller

GapFiller tries to fill any unclosed gaps in the draft genome using ultra-long sequences. This module takes the gap-tied draft genome alongside ultra-long sequences as input, all in FASTA format. First, the flanking sequences of gaps in the draft genome are extracted as anchors. After that, alignments are generated between these anchors and ultra-long sequences. After filtering the low-quality alignment, if a pair of anchors are aligned to the same sequence in a proper position, the sequence between them will be used to fill the corresponding gap. If multiple sequences meet the requirement above, the one that shows the highest homology with both anchors will be selected. This module adopts a conservative strategy and never modifies the raw sequence to avoid variation loss. Meanwhile, this module generates alignment using small flanking sequences, significantly reducing the time spent on alignment.

#### Module #3: TeloExplorer

TeloExplorer identifies telomeres in a given genome. This module only takes a complete genome in FASTA format as input. First, the most enriched telomeric-like repeat at the end of each chromosome is identified, and then the repeat distribution is detected. Finally, this module generates statistical information that reports the telomere repeat monomer and repeat times on each chromosome. The ‘explore’ and ‘search’ tools from the telomere identification toolkit (tidk) (https://github.com/tolkit/telomeric-identifier) are employed by this module with some improvements and adjustments. The ‘tidk explore’ tool sometimes misses telomere repeats that are less enriched and identifies non-telomeric repeats when the telomere is missing. The ‘tidk search’ tool generates telomeric repeat counts in each window, making it hard to read in text and requiring manual checks using the ‘tidk plot’ tool. TeloExplorer combines these tools to achieve more readable results and automatic workflow.

#### Module #4: CentroMiner

CentroMiner predicts centromere candidates in the genome independent of the experimental evidence. This module can take a genome in FASTA format alone as input. Optionally, inputting the transposable element (TE) annotation in GFF3 format may achieve better consequences. It is recommended to obtain this annotation using the extensive *de novo* TE annotator (EDTA) [21]. First, this module identifies all tandem repeat monomers and selects the monomers likely to be centromeric repeats on each chromosome based on period and copy number. These monomers are then clustered to reduce redundancy. After that, the representative monomers are aligned to corresponding chromosomes. Continuous matching regions are added to candidates and scored based on total match length and retrotransposon content. Finally, the candidate locations and main monomers are collected and reported. CentroMiner provides the first tool to predict centromere independent of experimental data or previous centromeric studies.

### Performance evaluation

#### The assembly of the HY4Q genome

To examine the performance of our workflow, we have adopted the entire modular functions of quarTeT to assemble and characterize the primary haplotype of *A. chinensis* cv. ‘Hongyang’. The same sequencing data and reference genome for Hongyang v4.0 assembly were used as the input [18]. A total of 616 176 264 bp primary contigs were assembled and phased by hifiasm [7]. Using Hongyang v3.0 [22] as the reference genome, AssemblyMapper assembled 605 014 655 bp contigs into 29 chromosomes in the draft genome, remaining 13 unclosed gaps. Then, GapFiller successfully filled 11 gaps with ONT ultra-long reads, generating a final genome of 606 235 533 bp with only two gaps, hereafter named HY4Q. The genome benchmarking universal single-copy orthologue (BUSCO) completeness and the LTR Assembly Index (LAI) of HY4Q were 98.9% and 16.22, respectively. TeloExplorer identified 23 pairs and six single telomeres, 52 in total. CentroMiner identified 11 366 representative centromeric-like repeat monomers, then predicted 841 possible centromere candidates on all 29 chromosomes (Table 1). The entire workflow spent approximately 35 minutes in wall clock time working on a server with four processors, or 8000 seconds in CPU time.

**Table 1:** The genomic feature of HY4Q and HY4P

**Genomic feature**	HY4Q	HY4P
Contig size (bp)	616 176 264	616 176 264
Genome size (bp)	606 235 533	606 055 014
Chromosomes	29	29
Mapping rate (%)	98.4	98.4
Gaps	2	0
Telomere (pair)	52 (23)	57 (28)
Centromere	29	29
BUSCO (%)	98.9	99.3
LAI	16.22	16.38

**Figure 2:**
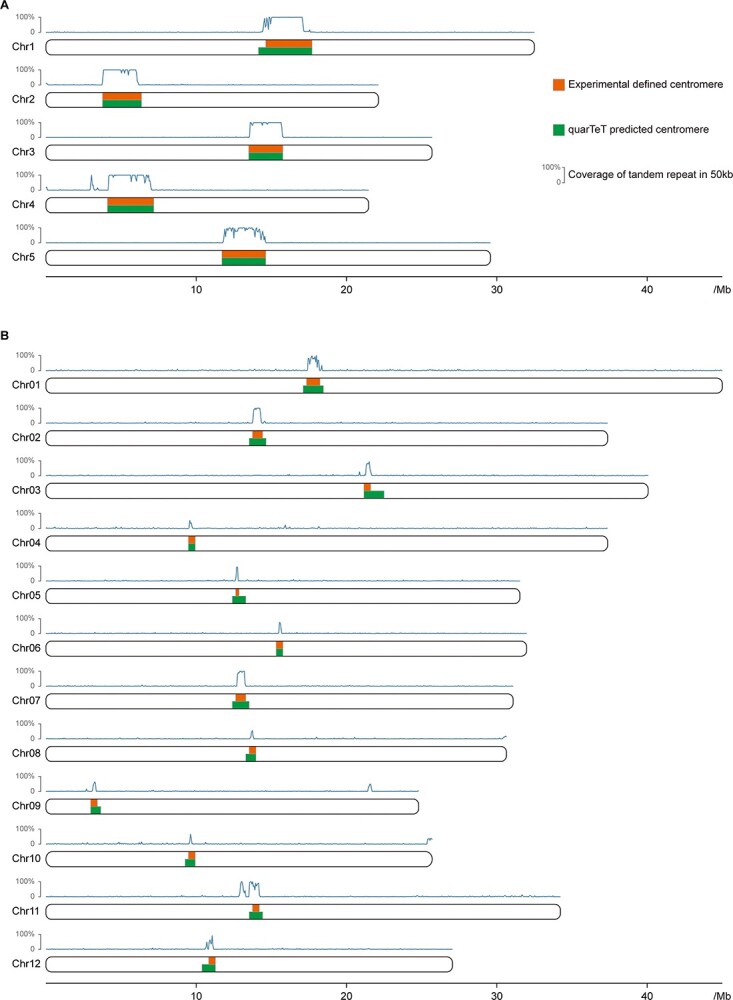
The centromeric region locations identified by the CentroMiner module in quarTeT. Centromeric regions identified by the quarTeT are colored in green, while centromeric regions located by experimental evidence are colored in orange. The line chart above each chromosome indicates the relative content of tandem repeats. **A***Arabidopsis thaliana* Col-CEN. **B***Oryza sativa* MH63RS3.

#### Centromere identification in *A. thaliana* and *O. sativa*

As the first tool to predict centromere independent of the experimental evidence, CentroMiner has been proven capable of identifying centromeric-like repeat regions. To further examine the accuracy of CentroMiner, we tried to identify centromeres in the Col-CEN assembly of *A. thaliana* [19] and *O. sativa* MH63RS3 genome [20], which have experimental evidence to locate centromeres. In the *A. thaliana* genome, CentroMiner identified the best centromere candidate on each chromosome, and these five candidates were all enriched with characterized 178 bp monomers *CEN180* (Fig. 2A). As for all 12 best centromere candidates identified in the *O. sativa* genome, 11 candidate regions were enriched with 155 ~ 165 bp *CentO* satellites. Only the best candidate predicted on Chr10 did not contain *CentO* satellites, but an over-enriched 148 bp repeat at the right end. However, enriched *CentO* satellites were found in the second-best candidate region on Chr10, though the content was poor compared to the centromeric regions on other chromosomes (Fig. 2B). This indicates that CentroMiner is efficient in identifying typical centromere independent of the experimental evidence.

## Discussion

With the development of TGS sequencing technologies and their corresponding analysis software, achieving a high-quality T2T genome assembly is no longer a big challenge [8]. However, tedious and time-consuming work is still required. Our group has focused on crop genomic study and recently assembled several T2T genomes, such as *A. chinensis* [18] and *Actinidia eriantha* [23]. In these previous works, we spent a long time in genome assembly. To benefit further studies, we developed many custom scripts for T2T genome assembly, which are integrated into the quarTeT toolkit. We also noticed the difficulty of studying the command line interface for beginners, so we tried to develop a user-friendly web toolkit to lower the learning cost.

**Figure 3:**
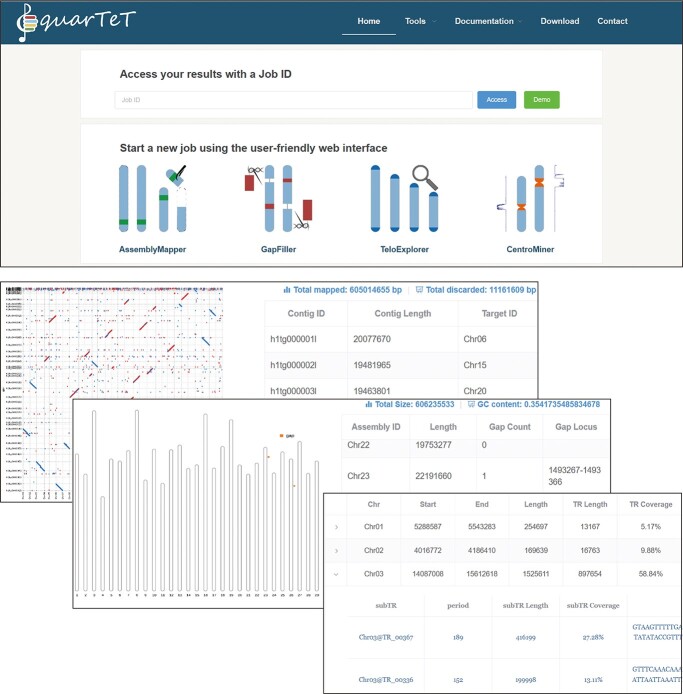
Presentation of the quarTeT web pages. The upper screenshot is a part of the home page of the quarTeT toolkit, including entries to modules, results, documentation, demo, etc. The lower screenshots are parts of the result pages, including collinearity, genome overview, and centromere candidates’ statistic.

Although we have optimized the workflow through various methods, catching up with the quality of manual assembly and characterization is still a great challenge. To evaluate the accuracy of the quarTeT toolkit, we compared HY4Q with the manually assembled and annotated genome of the same individual, the HY4P assembly [18]. In all 29 chromosomes, 17 chromosomes of HY4Q were identical to HY4P, and the other 12 chromosomes showed >99.97% identity, indicating that quarTeT is capable of assembling T2T genomes with high accuracy. The BUSCO completeness and LAI of HY4P were 99.3% and 16.38, only slightly better than HY4Q (Table 1). This indicates that the genome assembled by quarTeT could almost reach the quality of the manually assembled genome. Of all 29 centromeres defined in HY4P, 23 of them were identified as the top five candidates by CentroMiner. Only the centromere identified on Chr15 was inconsistent with HY4P. This demonstrates that the quarTeT is capable of identifying centromere in the T2T genome. HY4P genome is manually adjusted by combining several additional data, so it is hard to reach the same quality in an automatic workflow, though our toolkit still produces a high-quality genome assembly.

Among recently published genome assembly pipelines, the quarTeT toolkit is uniquely positioned. TRITEX has described a pipeline using ultra-long reads and Hi-C reads with open resource tools to assemble a high-quality genome [24], but it does not achieve an automatic workflow. The users are required to write a custom script in the Unix command line first, then manually adjust scaffolds using R prompt and Excel in the graphical user interface. In contrast, quarTeT allows an automatic workflow to assemble a T2T genome. RagTag provides a pipeline to assemble a high-quality genome with the aid of a reference genome and additional ultra-long sequences [25]. However, RagTag assumes that the reference genome is more reliable, and the sequences inconsistent with the reference are more likely to be considered errors. Nevertheless, the RagTag ‘patch’ tool applies an aggressive strategy that may discard variation or insert large segments to ultimately close the gaps. It also renames all sequences with an order not coincident with input, which often makes users confused. Conversely, the quarTeT toolkit adopts a conservative strategy and never modifies the raw sequence to avoid variation loss.

Meanwhile, the high variability and rapid evolution of centromere have puzzled many researchers in the studies of T2T genome. Although experimental methods can provide efficient evidence, the majority of researchers are unable to conduct the experiment due to the lack of instruments and reagents [14]. In most studies, the TRF program [17] is used to predict the centromeric repeat regions. However, a great challenge was opened to us when we tried this strategy on species in *Actinidia*. The TRF program failed to find a region enriched with tandem repeats for most chromosomes. To solve this issue, we deeply investigated the characterization of complex centromeric regions and finally developed a new method to identify the complex centromeres [18]. This method is provided in CentroMiner as the first tool to predict centromere candidates independent of experimental evidence or previous centromeric studies.

To ensure the general effectiveness and accuracy of this method, we examined the CentroMiner by identifying centromeres in *A. thaliana* and *O. sativa* genomes. The centromeres identified in the *A. thaliana* genome perfectly matched the regions reported in the previous study [19] (Fig. 2A). The centromeric regions identified in the *O. sativa* genome were slightly wider than the previous work defined [20], but the core regions were consistent (Fig. 2B). This indicates that the CentroMiner is capable of identifying centromere in most cases.

Compared to CentroMiner, other tools for centromere prediction depend on experimental evidence and previous studies. StringDecomposer can efficiently locate the centromeric regions in a given genomic sequence [26]. However, it requires known centromeric repeat monomers as input. Recently published HiCAT also faces the same issue [27]. These tools can only identify centromeres in well-studied species. Due to the lack of centromeric studies in previous decades, understanding of centromere is limited to several specific species [2]. In contrast, CentroMiner is able to identify centromeres in species that lack studies and discover the significant changes of centromeric repeat monomers.

The quarTeT is mainly designed to solve typical diploid genomes. When AssemblyMapper is used for polyploid genomes, the input contigs should be phased by appropriate programs, and the effectiveness depends on the phasing quality. Shortcomings should also be noticed when CentroMiner is used in complex genomes. Our method still requires manual checks to determine the centromere from candidates. This method sometimes scores unrelated repeat-rich regions higher than centromeric regions in the complex genome. Meanwhile, the flanking of the centromere may be overlooked. But with the rapid publishing of T2T reference genomes, the amount of centromere data resources is swiftly increasing. With a larger dataset, improving accuracy with an advanced algorithm based on big data would be possible. We are also trying to implement an algorithm based on machine learning to improve centromere identification in the near future.

In conclusion, quarTeT is a user-friendly web toolkit for studying T2T genome. It can assemble contigs into pseudo-chromosomes by referring to a reference genome, fill gaps with the aid of ultra-long sequences, identify telomeres, and predict centromere candidates. The quarTeT toolkit demonstrates the ability to assemble and characterize a high-quality T2T genome in an automatic workflow. The quarTeT toolkit is freely available at http://www.atcgn.com:8080/quarTeT/home.html (Fig. 3).

## Materials and methods

### Program and web server implementation

The command-line program of quarTeT is implemented in the Python3 programming language (https://www.python.org/). The web server is built on Apache Tomcat (https://tomcat.apache.org/) and implemented on a Linux Alibaba Cloud server with four cores and 8 GB RAM. The web pages are constructed using Spring Boot (https://spring.io/projects/spring-boot) and Vue (https://vuejs.org/). The data are stored in and served by a MySQL server (https://www.mysql.com).

### Dataset

The raw reads generated by HiFi and ONT platforms for the assembly of Hongyang v4.0 were downloaded from NCBI in accession of PRJNA869178 [18]. The complete genome of Hongyang v3.0 is downloaded from Kiwifruit Genome Database [28]. The complete Col-CEN genome assembly sequence of *A. thaliana* was downloaded from https://github.com/schatzlab/Col-CEN [19]. The complete *O. sativa* MH63RS3 genome sequence was accessed from NGDC in accession of PRJCA005549 [20].

### Data processing and visualization

The alignment between ultra-long sequences is generated by minimap2 v2.24-r1122 [6]. MUMmer v4.0.0rc1 [29] can also optionally replace minimap2 as the alignment tool. The telomeric repeats are localized by tidk v0.2.1 (https://github.com/tolkit/telomeric-identifier). The tandem repeat monomers are identified by Tandem Repeat Finder v4.09 [17] and clustered by CD-HIT v4.6 [30]. Local alignment between centromeric repeat monomers and the genome is generated by BLAST+ v2.8.1 [31]. The contigs for the assembly of HY4Q are assembled and phased by hifiasm v0.16.1 [7]. The BUSCO completeness of HY4Q is measured using BUSCO v5.4.3 [32]. The LAI of HY4Q is measured using LTR_retriever v2.9.0 [33]. The collinearity plot is generated by the ‘mummerplot’ script included in MUMmer v4.0.0rc1 [29]. The genome overview plot is generated by RIdeogram v0.2.2 [34], an R package (https://www.R-project.org/) running on R v3.6.0 and v4.2.2.

## Data Availability

The source code of the quarTeT toolkit command-line program is publicly available on GitHub (https://github.com/aaranyue/quarTeT). The dataset of HY4Q is available on the download page of the quarTeT website (http://atcgn.com:8080/quarTeT/download.html).
